# A systematic review of ayres sensory integration intervention for children with autism

**DOI:** 10.1002/aur.2046

**Published:** 2018-12-12

**Authors:** Sarah A. Schoen, Shelly J. Lane, Zoe Mailloux, Teresa May‐Benson, L. Dianne Parham, Susanne Smith Roley, Roseann C. Schaaf

**Affiliations:** ^1^ STAR Institute Greenwood Village Colorado; ^2^ School of Health Sciences, Faculty of Health and Medicine, Department of Occupational Therapy University of Newcastle Newcastle Australia; ^3^ Department of Occupational Therapy, Jefferson College of Health Professions Thomas Jefferson University Philadelphia Pennsylvania; ^4^ SPIRAL Foundation Newton Massachusetts; ^5^ University of New Mexico Albuquerque New Mexico; ^6^ Collaborative for Leadership in Ayres Sensory Integration Aliso Viejo California; ^7^ Department of Occupational Therapy Jefferson, College of Health Professions Faculty Farber Institute for Neurosciences Thomas Jefferson University Philadelphia Pennsylvania

**Keywords:** Sensory integration, occupational therapy, autism, treatment research, evidence‐based practice

## Abstract

Sensory integration is one of the most highly utilized interventions in autism, however, a lack of consensus exists regarding its evidence base. An increasing number of studies are investigating the effectiveness of this approach. This study used the Council for Exceptional Children (CEC) Standards for Evidence‐based Practices in Special Education to evaluate the effectiveness research from 2006 to 2017 on Ayres Sensory Integration (ASI) intervention for children with autism. A systematic review was conducted in three stages. Stage 1 involved an extensive database search for relevant studies using search terms related to sensory integration and autism, interventions suggesting a sensory integration approach, and high‐quality study designs. Searches yielded 19 studies that were evaluated in Stage 2. Six of these met inclusion criteria of being peer‐reviewed, written in English, description of intervention this is consistent with ASI intervention, and comparison group design or single subject method employed. Prior to analysis using CEC standards, three articles were excluded because intervention details were not consistent with the core principles of ASI, or because of major methodological flaws. In Stage 3, the remaining three studies were rated using the CEC quality indicators and standards for an evidence‐based practice. Two randomized controlled trials respectively met 100% and 85% of the CEC criteria items. One additional study met more than 50% of the criteria. Based on CEC criteria, ASI can be considered an evidence‐based practice for children with autism ages 4–12 years old. ***Autism Research*** *2019, 12: 6–19*. © 2018 The Authors. Autism Research published by International Society for Autism Research and Wiley Periodicals, Inc.

**Lay Summary:**

Ayres Sensory Integration intervention is one of the most frequently requested and highly utilized interventions in autism. This intervention has specific requirements for therapist qualifications and the process of therapy. This systematic review of studies providing Ayres Sensory Integration therapy to children with autism indicates that it is an evidence‐based practice according to the criteria of the Council for Exceptional Children.

## Introduction

Recently, standards for evidence‐based interventions in special education were published by the Council for Exceptional Children (CEC) to guide educators, therapists, researchers, parents, policy makers, and others to identify effective interventions and make informed decisions about practices for children receiving special education [Cook et al., 2015; Council for Exceptional Children [CEC], 2014]. The standards define and describe specific quality indicators (QIs) that can be used to evaluate intervention research, and are intended for use “by those with advanced training and experience in educational research design and methodology” to make decisions about evidence‐based interventions for children receiving special education services [Council for Exceptional Children [CEC], 2014, p. 5]. According to the CEC standards “a study is considered to have addressed a quality indicator when reviewers agree that the methodological issue is addressed satisfactorily such that it does not represent a meaningful threat to the validity of the study findings” [Council for Exceptional Children [CEC], 2014, p. 6]. Additionally, methodologically sound studies must meet the quality indicators relevant to their research design.

For children with autism spectrum disorders (ASD), occupational therapy is a frequently utilized service in special education that is designed to support a child's ability to access the educational curriculum and benefit from his or her education in the least restrictive environment [Hess, Morrier, Heflin, & Ivey, 2008; Wei, Wagner, Christiano, Shattuck, & Yu, 2014]. Occupational therapy using the principles of Ayres Sensory Integration® (ASI) intervention is among the most requested services by parents of children with ASD [Goin‐Kochel, Mackintosh, & Myers, 2009; Green et al., 2006; Mandell, Novak, & Levy, 2005], and is one of the most frequently utilized treatment approaches in pediatric occupational therapy [Case‐Smith & Miller, 1999; Mailloux & Smith Roley, 2010]. However, despite the high utilization of ASI for children who receive special education, there is lack of consensus regarding its evidence base. One reason for this is that many studies included in existing systematic reviews and meta‐analyses report on sensory‐based interventions which are not consistent with the principles of ASI as described by Ayres [Ayres, 1972, 1979, 1989; Ayres & Robbins, 2005] and operationalized in the Ayres Sensory Integration Fidelity Measure (ASIFM) [May‐Benson et al., 2014; Parham et al., 2007]. Instead, many reviews and meta‐analyses include studies of interventions that use isolated sensory stimuli as the active ingredient of the intervention (hereafter referred to as sensory‐based interventions) and do not adhere to the core principles of ASI [Barton, Reichow, Schnitz, Smith, & Sherlock, 2015; Lang et al., 2012]. These sensory‐based interventions are largely characterized as protocols that are passively applied to the child and have been found to have few positive effects [Case‐Smith, Weaver, & Fristad, 2015]. They lack many of the key ingredients of the ASI such as individual‐tailoring, active engagement of the child, the establishment of a therapeutic alliance between the child and therapist, targeting the just right challenge and provided within the context of play ([Parham et al., 2011]. In contrast, one recent review by Schaaf, Dumont, Arbesman, and May‐Benson [2018] that included only studies where “the intervention approach adhered to the principles of ASI” (p.3) concluded that ASI has strong evidence for positive outcomes on individual goals, moderate evidence supporting improvements in autistic behaviors and caregiver assistance for self‐care activities, and emerging but insufficient evidence for outcomes related to play, sensory‐motor skills, language, and social skills. Consequently, the conclusions of many of these prior reviews are inaccurate and misrepresentative.

ASI is an individualized intervention designed to address the specific underlying sensory‐motor issues that may be affecting children's performance during daily routines and activities, including participation within the classroom and in other contexts of the school. Application of ASI requires clinical reasoning to ensure that sensory‐motor activities address the specific difficulties identified in the assessment, and that these difficulties are linked to the child's functioning in daily life. The intervention takes place within a context of play, emphasizes active involvement of the child, involves a collaborative relationship between therapist and child, and focuses on participation‐oriented outcomes that are collected at regular intervals throughout the duration of the intervention program, making it possible to examine the child's response to intervention and to allow for adjustments to the intervention plan. Characteristics of the ASI intervention approaches have been delineated in textbooks as a guide to pediatric occupational therapy practice from 1972 to the present [Ayres, 1972, 1979; Ayres & Robbins, 2005; Bundy, Lane, & Murray, 2001; Case‐Smith & O'Brien, 2009; Kramer & Hinojosa, 2010; Lane & Bundy, 2012; Schaaf & Mailloux, 2015] and described in peer‐reviewed literature [May‐Benson et al., 2014; Parham et al., 2011; Schaaf, Benevides, Kelly, & Mailloux‐Maggio, 2012; Schaaf, Hunt, & Benevides, 2012]. Sensory‐based interventions stand in contrast to ASI in that their application often contradicts many of the core principles of ASI.

We have identified four major concerns with many of the past systematic reviews and meta‐analyses of studies claiming to evaluate sensory integration intervention. First, as noted above, past reviews typically included studies in which the “sensory integration” intervention described in the study was not consistent with the principles of ASI and was instead a sensory‐based intervention, i.e., it involved adult‐controlled application of sensory stimuli within a protocol that is provided in a similar way to all participants [Case‐Smith et al., 2015]. Individualization of intervention based on the child's assessment findings is absent or very limited, and passive cooperation is required of the child, rather than active collaboration with the therapist. This is particularly evident in Lang et al., 2012 and Barton et al., 2015 whose reviews included studies of the effects of isolated sensory‐specific strategies along with studies of sensory integration. Examples of such sensory‐based interventions highlighted in previous systematic reviews [Barton et al., 2015; Lang et al., 2012] include directing the child to wear a weighted vest, to sit on a therapy ball during classroom work, to accept sensory stimuli applied by the therapist in a specific protocol (such as brushing the skin or spinning the child on a rotating board), or to perform specified sensory‐motor activities or exercises [Case‐Smith et al., 2015;. Schaaf et al., 2018].

A second factor confounding interpretation of the evidence on ASI intervention is that past reviews and meta‐analyses include studies that failed to provide an adequate description of the phenotypic characteristics of participants [Barton et al., 2015; Lang et al., 2012; Weitlauf, Sathe, McPheeters, & Warren, 2017]. Of particular concern is failure to evaluate whether participants actually demonstrated specific sensory‐motor difficulties impacting performance and participation. For instance, Barton et al. [2015] indicate that only 20 of the 30 studies they included in their review measured sensory processing behaviors, and Lang et al. [2012] found that only 7 of the 25 papers included in this review had assessed sensory processing Consequently, characterization of the sample is poor, groups lack homogeneity, and individual tailoring of the intervention, a requisite of ASI, is missing. As a result, these studies may have evaluated interventions that were applied to children without the specific sensory‐motor difficulties that are appropriate for ASI intervention. Inclusion of these studies in systematic reviews and meta‐analyses makes it impossible to draw conclusions regarding efficacy of ASI intervention.

Third, many studies included in existing reviews do not present a replicable description of the intervention, nor do they document intervention fidelity throughout the intervention period using a quantitative measure [Devlin, Healy, Leader, & Hughes, 2010; Barton et al., 2015; Lang et al., 2012; Weitlauf et al., 2017] Thus, even for some studies that might have evaluated ASI intervention, it is unclear whether the intervention consistently followed the core principles of ASI and it is impossible to replicate the study.

Finally, outcomes measured in existing studies vary widely and may not be sensitive to the changes expected following ASI intervention. Varied outcomes across studies make it difficult to confidently synthesize findings of systematic reviews to identify the outcomes for which an intervention is likely to be most helpful [Case‐Smith & Arbesman, 2008; Case‐Smith et al., 2015; May‐Benson & Koomar, 2010; Watling & Hauer, 2015]. One previous review suggests that only measures of sensory and motor skills are impacted by this intervention [Weitlauf et al., 2017]. However, evidence of gains on broader family goals has been reported in the literature [May‐Benson & Koomar, 2010; Schaaf et al., 2014; Schaaf et al., 2018].

Given that the weakness in most prior reviews has interfered with an accurate appraisal of the evidence for ASI, the publication of the CEC standards presents an opportunity to critically evaluate the evidence in a rigorous and standardized way. Thus, the purpose of this paper is to utilize the CEC standards to determine whether ASI intervention meets the criteria for an evidence‐based practice for children with ASD. This systematic review differs from past reviews in that (1) the population is more narrowly defined (children with ASD between the ages of 4 and 12 years), (2) the intervention must meet a strict definition of ASI, and (3) the research question is specific to the evidence‐based criteria set forth by the Council for Exceptional Children. Unlike Weitlauf et al. [2017] and the most recent systematic review in the American Journal of Occupational Therapy [Schaaf et al., 2018], the current manuscript is targeted to professionals outside the field of occupational therapy to assist in decisions about referring children with autism to occupational therapists who use ASI intervention. Additionally, only the CEC standards and quality indicators (QIs) were used to determine which studies met the methodological features needed to assure confidence in study findings. According to the CEC standards “a study is considered to have addressed a quality indicator when reviewers agree that the methodological issue is addressed satisfactorily such that it does not represent a meaningful threat to the validity of the study findings” [Council for Exceptional Children [CEC], 2014, p. 6].

## Research Question and Design

This article addressed the question: Does ASI intervention meet the CEC criteria for an evidence‐based practice for children with autism spectrum disorders (ASD)? To answer this question, we conducted a systematic review of research studies that examined effectiveness of ASI intervention for children with ASD. We then analyzed the quality of each included study using the CEC Standards for Evidence‐based Practices in Special Education [Cook et al., 2015; Council for Exceptional Children [CEC], 2014].

## Methods

This systematic review was completed in three stages. The first stage involved a series of electronic database searches to locate potentially relevant studies. The second stage involved selection of studies using specific inclusion criteria related to methodology and description of the intervention. The third stage involved evaluation of included studies using the CEC standards [Cook et al., 2015] to determine whether ASI intervention meets the criteria for an evidence‐based practice for children with autism.

### *Stage One: Search Process*

In Stage One, searches were conducted in CINAHL, Cochrane Reviews, Cochrane Trials, Embase, ERIC, Medline, and PsychINFO databases, and initially included all citations from the inception of each database through May 2017. Next, we delimited our search to articles published after 2006 since this coincides with initial identification and articulation of the key structural and process elements of sensory integration intervention which became available for use by researchers and provide a guide for evaluating studies that meet ASI principles [Parham et al., 2007]. We searched literature through 2017.

Search terms addressed three broad content areas that were required of studies to be selected for analysis: condition, intervention, and study design. *Condition* refers to developmental conditions of study participants that suggest the presence of sensory integration problems. For this content area, we used search terms and Boolean phrases that are consistent with sensory integrative difficulties or sensory processing disorders, e.g., <sensory integrative disorders> OR <sensory processing disorders> OR <developmental dyspraxia>. The *intervention* content area refers to terms consistent with therapeutic strategies, tools, and constructs incorporated into ASI intervention. Examples of terms used for this content area include <Ayres Sensory Integration> OR <sensory integration> OR <motor planning> OR<play> OR <tactile stimulation>. The *study design* content area was operationalized using search terms such as <best practices>, <cohort studies>, <case control>, and <randomized controlled trial>. Search terms that produced articles within each content area were combined using the Boolean operator “AND,” and searches were limited to English language as well as participants whose age range fell within 0–18 years. Search terms and structure varied to some extent across databases due to unique requirements of each database. The strategy used in Medline is presented in the Search Strategy for Medline Table, found in supplemental materials.

**Figure 1:**
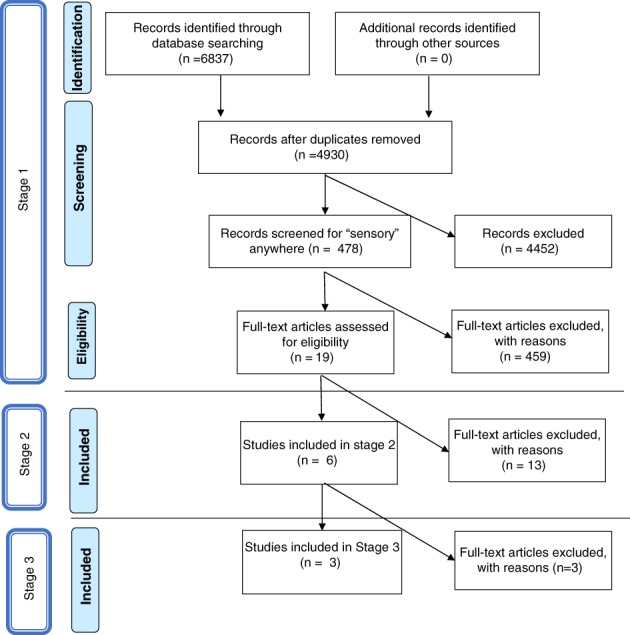
Search strategy.

An iterative process of record reduction was conducted using the above search process. The number of references retrieved from each database is presented in Table 1. A PRISMA diagram summarizing the result of each step of the search process is shown in Figure 1. A total of 6,837 references were retrieved initially, reduced to 4,930 after removing duplicates. An initial filter searching for “sensory” anywhere in the retrieved records excluded an additional 4,452 references, leaving 478 records for title and abstract review. Title and abstract review resulted in exclusion of 459 articles that did not meet the following criterion, which addresses all three key content areas: an *intervention study* that addresses *sensory issues* of *children with autism*. After the initial filtering, two authors (SAS and SJL) reviewed article titles, abstracts, and when necessary, full texts, to screen for articles that met the criterion. Additionally, hand searching was conducted of reference lists from final articles to ensure thoroughness; no additional articles were identified that fully met the criterion. At the end of Stage One screening, 19 studies remained.

**Table 1:** Databases Searched and Records Identified

Database	Records identified	Number of records after de‐duplication
CINAHL	1058	641
Cochrane Reviews	5	3
Cochrane Trials	342	131
Embase	2057	1530
ERIC	310	152
Medline	1096	1029
PsycINFO	1969	1444
Total	6837	4930

**Table 2:** Studies Excluded from CEC Analysis

Article	Reason(s) for exclusion	Comments
Sankar [2015]	Not ASI	Ambiguity regarding intervention employed. Authors described intervention as “sensory integration” and provide examples of several activities consistent with this approach, however, there is insufficient evidence that ASI principles were followed. No fidelity, no training of interventionists, no statistics reported
Dunbar, Carr‐Hertel, Lieberman, Perez, and Ricks [2012]	Study Type; Descriptive	Ambiguity regarding methodology employed. Descriptive study with no statistical analyses employed
Piravej, Tangtrongchitr, Chandarasiri, Paothong, and Sukprasong [2009]	SI included In both groups	Authors described intervention as “sensory integration therapy,” however, because both groups received SI (e.g. SI *vs* Massage & SI) effects of SI alone could not be judged

*Note*. Exclusion Criteria. IQ = Participants IQ not below 65. Diagnosis = Participants did not have Autism Spectrum Disorder. Study Type = Study was not a group comparison or appropriate single‐subject design examining effectiveness of ASI. Not ASI = Intervention did not meet criteria for Ayres Sensory Integration© intervention. ASI = Ayres Sensory Integration© intervention.

### *Stage Two: Study Selection*

In Stage Two, the 19 studies were further examined to determine if they met the following criteria: published in the peer‐reviewed scientific literature; written in English; consistent with ASI theory; and utilized a group comparison design (with or without random assignment) or a multiple baseline, changing criterion, or alternating treatment single case experimental design. ABAB reversal designs were not included because the removal of ASI intervention is not expected to produce a return to baseline performance or behavior. Consistency with ASI intervention principles was determined by examining the description of the intervention to determine its adherence to the principles of ASI as described in seminal works such as Ayres [1972, 1979], Bundy et al. [2001]; Kramer and Hinojosa [2010]; Parham et al. [2011]. Evidence of adherence to the principles described in the ASI Fidelity Measure was considered although the specific use of the measure was not required in order for the study to be included in the Stage Three review process. Furthermore, at this stage, studies were required to include participants with a diagnosis of autism spectrum disorder (ASD) or pervasive developmental disorder (PDD) who had an IQ above 65. This final criterion was established because the principles of ASI often have to be modified for children with ASD who have lower IQs [Mailloux, Blanche, & Schaaf, 2001]. Studies were excluded if the intervention was sensory‐based, as discussed earlier, or if insufficient information was provided to determine adherence to ASI core principles.

At the end of Stage Two, six articles remained. In Stage Three, these six articles were subjected to a detailed analysis based on the CEC Quality Indicators.

### *Stage 3: Data Analysis Using CEC Criteria*

Raters who participated in the CEC review process were seven occupational therapy researchers (authors of this manuscript) with advanced‐level expertise in occupational therapy and sensory integration. Experience ranged from 34 to 42 years; all held an academic position and four of the seven were also active in clinical practice. All of the reviewers had experience in designing and conducting research, had completed advanced training in sensory integration including theory and intervention, and were trained in the use of the ASI Fidelity Measure. To reduce the risk of bias, each of the six remaining articles was reviewed and rated independently by at least two reviewers.

Three of the six reviewed papers were excluded from further analysis because the intervention description was inconsistent or insufficient to be confidently considered ASI intervention, or because of significant methodological concerns. Of note, all three studies that were excluded reported positive results for the intervention. The remaining three articles were subjected to full analysis using the CEC standards to evaluate whether ASI can be considered an evidence‐based intervention.

The CEC Standards for Evidence‐Based Practices in Special Education [Cook et al., 2015] are a set of evidence‐based practice standards that can be used by qualified researchers to evaluate intervention studies. They include eight quality indicators (QIs) which address context and setting, participants, intervention agent, description of practice, implementation fidelity, internal validity, outcome measure/dependent variables, and data analysis. Each of the eight QIs is operationalized using specific criteria that can be rated to evaluate the methodological rigor of an intervention research study. The completed ratings of QI criteria across multiple studies can then be used to classify a special education practice as falling into one of the following categories: evidence‐based, potentially evidence‐based, mixed evidence, insufficient evidence, or negative effects.

No reviewers rated a study in which they were an investigator, author, or consultant. Bias was minimized by strict adherence to the CEC QI criteria, as well as advisement from a CEC Standards author (B. Cook, personal communication, August 26 and September 22, 2016) to clarify interpretation of QI details. The reviewers independently provided a yes or no rating for each QI criterion and wrote a short summary justifying each rating. Occasional discrepancies between raters were discussed and resolved using a modified Delphi process.

The three studies that met all inclusion criteria and were included in the final analysis are summarized in Table 3, which is a composite table of QI ratings for each study, as well as justifications for each rating. Next, the CEC criteria for classifying a practice as evidence‐based were applied. These criteria are shown in Table 4.

**Table 3:** CEC Quality Indicator Ratings for Reviewed Studies

CEC QI Criterion	Iwanaga et al. [2014]		Pfeiffer, Koenig, Kinnealey, Sheppard, and Henderson [2011]		Schaaf et al. [2014]	
	Rating	Comments	Rating	Comments	Rating	Comments
*1. Context and setting*	The study provides sufficient information regarding the critical features of the context or setting relevant to the review; for example, type of program or classroom, type of school, curriculum, geographic location, setting, etc.					
Describes critical features of context or setting relevant to review, including type of program or classroom, type of school/facility, curriculum/intervention geographic location, community setting socioeconomic status, and, physical layout.	Yes	Out‐patient OT program (ASI and group social skills training) provided in specified medical/education center and university clinics in Nagasaki Japan; similar SES by proxy as subjects came from Nagasaki; description of SI clinic with fidelity requirements noted	Yes	Supplemental OT services provided at rural summer camp in Pennsylvania; similar SES by proxy as subjects attended same community‐based camp program; ASI provided in 3 SI rooms that met ASI fidelity requirements and OT‐fine motor group provided in 1 fine motor room	Yes	Out‐patient OT program at children's hospital in a New Jersey community provided ASI; SES reported by proxy *via* education level (largely college educated); equipment and space met ASI fidelity standards
*2. Participants*	Provides sufficient information to identify population of participants to which results may be generalized and determine or confirm whether participants demonstrated disability or difficulty of focus.					
2.1 Describes participant demographics relevant to the review including characteristics such as gender, age/grade, race/ethnicity, socioeconomic status, language status, etc. 2.2 Describes disability or risk status of the participants and method for determining status.	Yes	2.1 Subjects primarily males, ages 2.9–6.2 years; race/ethnicity presumed Japanese; language status not relevant but IQ scores >70. 2.2 ASD diagnosis per DSM‐IV; information from clinical records and pediatrician diagnosis; sensory integrative disorder identified by JMAP; participant ages appropriate for measures; IQ scores determined using appropriate tool; specified subjects attended regular nursery school or kindergarten, had no previous therapy and parents/teachers, etc. had no previous knowledge of sensory integration	Yes	2.1 Subjects primarily males, ages 6–12 years; language status not relevant but ASD diagnosis per reported DSM‐IV criteria. SES and race/ethnicity not included. 2.2 Autism diagnosis verified by report from qualified diagnosticians; sensory integration deficits identified by SPM and comprehensive evaluation by an expert ASI clinician	Yes	2.1 Subjects primarily males, 4.0–8.11 years, primarily Caucasian; cognitive level reported; language status not relevant. Parent level of education as an estimate of SES. 2.2 ASD diagnosis and autism severity identified by licensed psychologist using ADI‐R and ADOS; sensory integration difficulty identified by scores on SP and SIPT
*3. Intervention agent*	Provides sufficient information regarding critical features of intervention agent.					
3.1 Describes role of intervention agent and relevant background variables. 3.2 Describes any specific training or qualifications required to implement the intervention, and indicates that the interventionist has achieved them.	Yes	3.1 Group therapy provided by occupational therapist, speech therapist and 3 nursery school teachers to small group of 5–6 children; first author (OT) administered JMAP evaluations and SI treatment 3.2 Interventionist OT certified by Japanese Sensory Integration Association; group interventionists backgrounds not stated.	Yes	3.1 Fine motor group provided by OT graduate students, with supervision; ASI intervention provided by OTs; at least one treatment session/child was video recorded and scored for fidelity to ASI supporting appropriate role for interventionist 3.2 Evaluators and interventionists received training before implementing intervention including principles from ASI fidelity measure	Yes	3.1 ASI intervention provided by licensed OTs (n = 3; mean years of experience = 15, range 12–20 years), experienced working with children with ASD; evaluators (blinded to conditions) were also licensed OTs; comparison group was usual care as reported by parents 3.2 Interventionists and evaluators completed certificate programs in ASI including use of the SIPT
*4. Description of practice*	Provides sufficient information regarding critical features of practice (intervention); practice is clearly understood and can be reasonably duplicated					
4.1 Study describes detailed intervention procedures such as instructional behaviors, critical or active elements, manualized or scripted procedures, dosage, intervention agents' actions, or cites one or more accessible sources that provide this information. 4.2 When relevant, study describes materials (e.g., manipulatives, worksheets, timers, cues, toys), or cites one or more accessible sources providing this information.	Yes	4.1 Practice described relative to components of ASI fidelity measure (which was not available at time of this study); dosage 1 hr/week for 8–10 months (average of 9.3 months; no specific manual, but intervention followed 10 key therapeutic strategies of ASI Group therapy dosage 1.5 hr/week for 8–10 months (average 9.3 months); activities described briefly (social skill training, communication training, kinetic activities, child–parent play); environment not described 4.2 Provides description of materials, activities and additional resources for ASI intervention	Yes	4.1 Practice described as SI intervention based on ASI fidelity measure and grounded in Ayres SI treatment theory; dosage 18 sessions, 45 min each for 6 weeks, one child received 17 sessions; intervention followed 10 key therapeutic strategies for ASI Fine motor treatment training and intervention based on fidelity measure developed for this study; Basis for treatment for both groups described and examples provided with literature resources cited 4.2 Specific materials not described but general activities for both groups presented with additional resources provided for ASI intervention	Yes	4.1 Practice well described as ASI; dosage 3 hr/week, 1‐hr sessions, total of 30 sessions over 10–12 weeks; specific details of intervention provided with reference to manual pending publication at time of study; multiple appropriate sources for more information cited; adherence to fidelity conducted in a feasibility study prior to study and results published elsewhere 4.2 Examples and description of some equipment/materials and f specific activities with multiple appropriate sources for more information
*5. Implementation Fidelity*	Practice is implemented with fidelity.					
5.1 The study assesses and reports implementation fidelity related to adherence using direct, reliable measures (e.g., observations using a checklist of critical elements of the practice). 5.2 The study assesses and reports implementation fidelity related to dosage or exposure using direct, reliable measures (e.g., observations or self‐report of the duration, frequency, curriculum coverage of implementation). 5.3 As appropriate, the study assesses and reports implementation fidelity (a) regularly throughout implementation of the intervention, and (b) for each interventionist, each setting, and each participant or other unit of analysis.	No	5.1 Study refers to principles of ASI, but does not use fidelity measure 5.2 Indicates dosage, but does not use a fidelity measure 5.3 N/A since fidelity not measured	Yes	5.1 ASI fidelity measure used; all ASI sessions met fidelity criteria of >80; fine motor fidelity measure developed for study including 3 main focus areas, fidelity criteria, and score range to support assessment of fidelity; all fine motor sessions met fidelity criteria of ≥75 5.2 Dosage indicated by session length, number of sessions/week; total number of sessions; subjects completed 18 sessions, except one subject completed 17 5.3 All sessions checked for fidelity	Yes	5.1 Manualized intervention with adherence to fidelity; reported psychometrics for fidelity measure; all intervention sessions recorded; 10% evaluated and rated for fidelity 5.2 ASI group received all scheduled sessions; usual care group reported weekly services received; total weekly services for both groups reported; no significant differences reported between groups for interventions other than ASI; Attrition reported for both groups; 5.3 10% randomly selected ASI sessions subjected to fidelity checks throughout study, allowing changes to be made to insure fidelity
*6. Internal validity*	Independent variable is under control of experimenter. Study describes services provided in control and comparison conditions and phases. Research design provides sufficient evidence that independent variable causes change in dependent variable or variables. Participants stayed with study, so attrition is not a significant threat to internal validity					
6.1. The researcher controls and systematically manipulates the independent variable. 6.2. The study describes control/comparison conditions, such as the curriculum, instruction, and interventions. 6.3. Control/comparison‐condition or baseline‐condition participants have no or extremely limited access to the treatment intervention. 6.4. The study clearly describes assignment to groups. 6.8. Overall attrition is low across groups (e.g., < 30% in a 1‐year study). 6.9. Differential attrition between groups is low (e.g., ≤10%) or is controlled for by adjusting for noncompleters.	No	6.1 Nonrandom group assignment; retrospective record review study; intervention not prospectively controlled 6.2 Control condition described and reported 6.3 ASI intervention not provided to control group 6.4 Nonrandom group assignment with comparisons between group on multiple factors; no significant differences between groups; ASI group assignment depended on group therapy being full; group therapy did not receive ASI; no discussion or consideration of potential factors that may have influenced outcomes, aside from potential maturation effects 6.8 Subject selection process from existing records reported; 24 subjects met inclusion criteria, 4 (<20%) removed due to some invalid item scores on pre–posttests 6.9 10% difference in n between groups, unspecified but likely due to loss of subjects due to invalid test items.	Yes	6.1 Intervention under experimenter control 6.2 Researchers and caregivers blind to group membership 6.3 ASI intervention provided and available only through camp‐based study 6.4 Random assignment to ASI or fine motor groups by statistician not associated with study using SPSS 6.8 10% attrition; report of 4 “drop outs”, group not specified; 4 other children removed by experimenters or parents 6.9 10% attrition, groups not reported but smaller n in fine motor group assume losses mostly in that group	Yes	6.1 Intervention under control of investigator 6.2 Control condition described and reported; additional services for both groups adequately recorded weekly and reported 6.3 Both groups received equal amounts of school‐based OT but only intervention group received ASI 6.4 Random assignment to treatment or usual control group by random permuted blocks within four strata based on cognitive level with controls for IQ and autism severity; no significant differences in preintervention demographic criteria; 6.8 Attrition less than 10%; 6.9 No attrition in intervention group; control group lost 1 to posttesting and 1 completed partial follow‐up
7. *Outcome measures/dependent variables*	Outcome measures are applied appropriately to gauge effect of the practice on study outcomes. Outcome measures demonstrate adequate psychometrics.					
7.1. Outcomes are socially important in that, they constitute or are theoretically or empirically linked to improved quality of life, an important developmental/ learning outcome, or both. 7.2. Study clearly defines and describes measurement of dependent variables 7.3. Study reports effects of intervention on all measures of outcome targeted by the review (*p* levels and effect sizes [ES]) or data from which ESs can be calculated for group comparison studies. 7.4. Frequency and timing of outcome measures are appropriate. 7.5. Study provides evidence of adequate internal reliability, inter‐observer reliability, test–retest reliability, or parallel form reliability, as relevant. 7.6. Study provides adequate evidence of validity, such as content, construct, criterion (concurrent or predictive), or social validity.	No	7.1 Outcomes related to sensory, motor, cognitive measures are socially appropriate 7.2 No psychometrics presented; components of interest on measure described 7.3 Sufficient data presented to calculate effect sizes 7.4 Frequency and timing of outcome measures appropriate 7.5 No psychometric information on outcome measures reported 7.6 No psychometric information reported	Yes	7.1 Socially important GAS goals, SRS, and other outcomes. 7.2 Measures clearly described; psychometrics presented for outcome measures 7.3 All outcomes reported or data available to calculate effect sizes 7.4 Frequency and timing of outcome measures appropriate 7.5 Reliability for outcome measures reported 7.6 Validity presented when available	Yes	7.1 Goals socially appropriate, GAS goals individualized to child quality assurance of GAS goals established; other functional outcomes based on literature 7.2 Measures clearly described and strength of psychometrics detailed for all outcome measures 7.3 All measures included in results; p levels and effect sizes presented 7.4 Frequency and timing of outcome measures appropriate 7.5 Reliability for outcome measures reported 7.6 Evidence of validity of outcome measures reported
*8. Data analysis*	Data analysis is conducted appropriately. Study reports information on effect size (ES)					
8.1 Data analysis techniques are appropriate for comparing change in performance of two or more groups. 8.3 Study reports one or more appropriate effect‐size statistics for all outcomes relevant to review being conducted, even if the outcome is not statistically significant, or provides data from which appropriate ESs can be calculated.	Partial	8.1 Analysis techniques appropriate for comparing change; large number of analyses for small sample 8.3 Sufficient data presented to allow calculation of effect sizes. Partial η^2^ calculated for JMAP. Effect size average below 0.25.	Partial	8.1 Use of statistical consultant; analysis appears appropriate for data 8.3 Partial η^2^ calculated effect sizes on all measures; effect sizes on GAS and autistic mannerisms on SRS reported; sufficient data reported to calculate effect sizes on other measures. Effect size average below 0.25	Yes	8.1 Analysis techniques appropriate for date; secondary outcomes nonnormally distributed and some nonsignificant differences between baseline scores of groups might be considered clinically relevant justified use of change scores rather than standard scores 8.3 Effect size presented for GAS, PEDI change scores, and PDDBI change scores. Effect size average above 0.25

*Note*. Items 6.5, 6.6, 6.7, and 8.2 removed from the table as they applied only to single‐subject studies.

UC = Usual Care; SES = socio‐economic status; SI = sensory integration; ASI = Ayres Sensory Integration; GAS = Goal Attainment Scaling; PEDI = Pediatric Evaluation of Disability Inventory; PDDBI = Pervasive Developmental Disorders Behavioral Inventory; SPM = Sensory Processing Measure; SRS = Social Responsivity Scale; SPSS = Statistical Package for the Social Sciences; JMAP = Japanese Miller Assessment for Preschoolers.

**Table 4:** Council for Exceptional Children Evidence‐Base Classifications of Practices in Special Education

CEC Criteria	
To be considered an evidence‐based the practice must meet either A or B	
A	B
The practice must be supported by two methodologically sound group comparison studies with random assignment to groups, positive effects, and at least 60 total participants across studies; OR Four methodologically sound group comparison studies with nonrandom assignment to groups, positive effects, and at least 120 total participants across studies; OR Five methodologically sound single‐subject studies with positive effects and at least 20 total participants across studies.	Meet at least 50% of criteria for two or more of the study designs described in A; AND Include no methodologically sound studies conducted with negative effects and at least a 3:1 ratio of methodologically sound studies with positive effects to methodologically sound studies with neutral/mixed effects (includes group experimental, nonrandomly assigned group comparison, and single‐subject design studies collectively).

Adapted from *“*Council for Exceptional Children Standards for Evidence‐Based Practices in Special Education*”* by Cook et al., 2015, *Remedial and Special Education*, *36*, 220–234. Copyright 2014 by the Council for Exceptional Children.

For experimental group comparison studies, the CEC Standards use effect size rather than statistical significance to evaluate the strength of the findings, since statistical significance is influenced by the sample size. Effect size is preferable because it takes into account the meaningfulness of the outcomes for the population being studied. Therefore, the final three studies were analyzed using the suggestion of the CEC Standards authors to use the guidelines of the What Works Clearinghouse [2011], where an effect size ≥0.25 is deemed a substantively important intervention effect and <0.25 is not a substantively important effect.

## Results

The ratings and comments for the three articles that met the inclusion criteria are presented in Table 3. These studies are presented in alphabetical order below.

Iwanaga et al. [2014] is a nonrandomized study that compares outcomes data from children with ASD who received 9 months ASI intervention (*n* = 8) to those who received 9 months of group therapy (social skills training, communication training, kinetic activities, and parent–child play; *n* = 12). The context and setting are a university‐affiliated medical center in Japan where a majority of sessions were held for both groups. Participant age and diagnosis are described for each group, but not SES or race/ethnicity. Children were diagnosed with ASD according to DSM‐IV criteria [American Psychiatric Association, 2000]; IQ scores were above 70. The mean age of participants in each group was 4 years 8 months at the beginning of intervention, and 5 years 6 months at the end of intervention. ASI intervention was administered by an occupational therapist with advanced training in ASI. The group therapy was administered by a team of therapists and educators; however, their specific qualifications are not presented. ASI intervention was implemented in a therapy room with equipment consistent with ASI (e.g., a swing, ball pool, ladder, or trampoline). The intervention process is adequately described and clearly is consistent with the principles of ASI. The key features of the comparison treatment are also delineated, but fidelity checks are not reported for either group. The outcome measure is the Japanese re‐standardization of the Miller Assessment for Preschoolers (JMAP; Miller, 1982; Tsuchida, Sato, Yamada, & Matsushita, 1989). It provides sensory, motor, verbal, nonverbal, complex tasks, and total scores. Results indicate positive and statistically significant gains for the ASI group on five of the six outcome measures; effect sizes are not reported but can be calculated from the data. The average effect size that was calculated using eta squared is 0.23, which is below the 0.25 cutoff recommended by the What Works Clearinghouse [2011] guidelines.

Pfeiffer et al. [2011] is a randomized controlled trial comparing two occupational therapy interventions for children with ASD: ASI (*n* = 20) and fine motor training (*n* = 17). Children in each group received eighteen 45 min intervention sessions over a 6‐week period during a summer therapeutic activities program. Critical features of context and setting are described sufficiently, and the study provides demographic details on participant age and gender, however, no information is provided on socioeconomic status or race/ethnicity. Authors indicate participants had been diagnosed with ASD using DSM IV criteria, based on reports provided by a qualified diagnostician. Ages were 6–12 years old, with a mean age of 8.8 years at entry. Sensory integration difficulties were confirmed for all participants through a complete evaluation prior to beginning intervention. Implementation fidelity throughout the study is assured through the use of the ASI Fidelity Measure [Parham et al., 2011] for the ASI group, and a fine motor training fidelity measure developed for the comparison group. Interventionists were trained in their respective interventions *via* didactic training aligned with the appropriate fidelity measure. Researchers and raters were blinded to group membership. Four children did not complete the study, and one received one less treatment session than all others. The primary outcome measure (Goal Attainment Scale [GAS]) is socially relevant, as goals were set by parents and teachers, and were measured according to best practice guidelines for GAS. Findings indicate both groups made statistically significant improvements, but the ASI group showed greater improvement on GAS goals, as well as a significant decrease in autism mannerisms as measured by the Social Responsiveness Scale (SRS) [Constantino & Gruber, 2012]. Effect sizes for ASI indicate positive effects for GAS goals (effect size *=* 0.360 for teacher ratings and 0.125 for parent ratings), as well as for autism mannerisms on the SRS (*d* = 0.131). However, the average of these three effect sizes is 0.21, which is below the 0.25 cutoff recommended by the What Works Clearinghouse [2011] guidelines.

Schaaf et al. [2014] is a randomized controlled trial of 32 children with ASD, 6–9 years old, who received either usual care or ASI intervention for 30 one‐hour sessions over a 10‐week period. Randomized blocks were used to maximize equivalence of groups for cognitive status and severity of ASD. The context, setting, socioeconomic (SES) background, and physical layout of the intervention are described adequately, as are participant demographic characteristics, including age, race/ethnicity, and gender, to ensure replicability. An accepted estimate of SES is report of parent education level. This study uses the ADIR and ADOS to confirm diagnosis of ASD, and independent, blinded evaluators trained to administer outcome measures. The study describes manualized training of the interventionists to provide ASI intervention with strong fidelity. Fidelity was monitored regularly throughout intervention with the number of fidelity checks reduced as fidelity met criterion level. All participants assigned to ASI received a full assessment of sensory integration, enabling interventionists to individualize treatment. The description of the ASI treatment environment is consistent with fidelity guidelines [May‐Benson et al., 2014]. Internal validity is strengthened by documentation that the “usual care” received by the study group and control group was equivalent. Attrition is minor (*n* = 2) and not a significant threat to internal validity. The primary outcome measure, GAS, is socially valid and was administered using rigorous methods [Krasny‐Pacini, Evans, Sohlberg, & Chevignard, 2016]. The secondary outcome measure, the Pediatric Evaluation of Disability Inventory (PEDI) [Haley, Coster, Ludlow, Haltiwagner, & Andrellos, 1992] has strong psychometric properties. Results indicated statistically significant group differences favoring the ASI group. A very large effect size (Cohen's *d =* 1.20) for GAS outcomes, and large effect sizes for caregiver assistance on PEDI self‐care (*d* = 0.9) and social activities scales (*d* = 0.7) are reported for the ASI group. The average effect size is 0.933, well above the What Works Clearinghouse [2011] guidelines.

## Discussion

Our study indicates that ASI intervention meets criteria for an evidence‐based practice for 4–12 year old children with autism, according to the CEC *Standards for Evidence‐Based Practices in Special Education* [Cook et al., 2015], which states, “meets at least 50% of criteria for two or more of the study designs described in (a)” (p. 9). The age range presented in these studies is inclusive of children who are typically referred to occupational therapy for ASI intervention. This determination that ASI is an evidence‐based practice is supported by the finding of two methodologically sound group comparison studies with random group assignment, positive outcomes, and a collective total of >60 participants. Specifically, Pfeiffer et al. [2011] met over 85% and Schaaf et al. [2014] met 100% of the CEC methodological quality indicators (QIs). Both studies were randomized clinical trials, had positive outcomes, and collectively had a total of 69 participants. Effect size averages across measures in the Schaaf study were well above the What Works Clearinghouse [2011] threshold for a desirable effect size, recommended by authors of the CEC Standards as a starting point for considering whether intervention effects are meaningful. Pfeiffer study effect sizes were slightly below the 0.25 cutoff. A third study [Iwanaga et al., 2014], although less rigorous in design, met 50% of the CEC criteria and was consistent in detecting positive, statistically significant outcomes of ASI intervention for children with ASD. Although ASI intervention may be appropriate for a wide range of children with ASD, results of this evidence‐based review apply only to children with ASD who have IQs above 65.

The intervention frequency varied across studies; Pfeiffer participants received therapy three times per week for 6 weeks, Schaaf study participants received therapy three times per week for 10 weeks and for the Iwanaga study participants received therapy once a week for 36–40 weeks. This range, from 18 to 40 sessions, is not uncommon in pediatric occupational therapy practice as there are no definitive guidelines to guide dosage. Positive results for even the lower dose of therapy suggest the need for further study of an optimal frequency and intensity of intervention. Effect sizes, while respectable, also varied across studies because each used a different effect size calculation; Schaaf et al. [2014] used Cohen's *d,* Pfeiffer et al. [2011] used a partial η^2^ and *r* was calculated by these reviewers for the effect size of the Iwanaga et al. [2014] study.

Although this study focused on the CEC Guidelines, findings here are supported through application of other published guidelines as well. The United States Preventative Services Task Force [2012] criteria for evidence‐based practices make the designation of *strong evidence* if there are at least two methodologically‐sound randomized controlled trials with consistent findings from these studies. The Pfeiffer et al. [2011] and the Schaaf et al. [2014] studies meet the U.S. Preventive Services Task Force criteria for strong evidence.

Similarly, application of the Frank Porter Graham (FPG) Child Development Institute criteria for evidence‐based practices for autism [Wong et al., 2015] shows that ASI meets these criteria as well. Using the FPG Child Development Institute standards, ASI is classified as an evidence‐based practice, as it is supported by two high‐quality randomized trials conducted by two different research groups.

Since completion of this review, one additional study of “sensory integration” was published [Kashefimehr, Kayihan, & Huri, 2018]. This study was not included in the current review because it was published after the inclusion criteria date range. Interesting, although not subjected to a full review, this study clearly meets many of the CEC quality indicators described earlier. For example, this was a randomized controlled trial of children with autism who received intervention consistent with the principles of ASI and had positive outcomes that impacted their participation in daily life activities and routines.

The findings from this review diverge from those of earlier reviews [Case‐Smith & Arbesman, 2008; Case‐Smith et al., 2015; May‐Benson & Koomar, 2010; Watling & Hauer, 2015]. However, this review is unique compared to past reviews of sensory integration intervention in several ways. The studies included in this review used a manualized approach reflected by references within the papers to occupational therapy textbooks and chapters that delineate the key characteristics and use of ASI intervention. Additionally, the ASI Fidelity Measure which was used by Schaaf et al. and Pfeiffer et al. assures that these studies met criteria for ASI intervention whereas previous reviews often included studies using sensory‐based interventions that did not meet the criteria for ASI [Barton et al., 2015; Lang et al., 2012]. Use of the ASI Fidelity Measure in those two studies enabled the authors to avoid a problem that characterizes most studies of ASI effectiveness: the conflation of diverse sensory‐based interventions with ASI intervention, making it impossible to draw conclusions about the effectiveness of ASI intervention [Case‐Smith et al., 2015].

Use of the ASI Fidelity Measure, or careful comparison to the core principles of ASI, is necessary to ensure that interventions purported to be ASI, are indeed consistent with the core principles. For example, a study conducted by Devlin et al. [2010] concluded that behavioral interventions are more effective than “sensory integration therapy” in reducing challenging behaviors of children with autism. However, comparison of the “sensory integration therapy” procedures in this study to the key elements of ASI, as delineated in the ASI Fidelity Measure, indicates that many critical ingredients of ASI intervention were missing. For example, the interventionist did not tailor challenges to assure they are slightly beyond the child's current level of performance (e.g. the just right challenge), did not collaborate with the child in activity choice, and did not ensure that the child experienced success. Although the study reports that the therapy was designed by an occupational therapist trained in sensory integration, the intervention described in the article was not consistent with ASI intervention principles [Case‐Smith et al., 2015]. Therefore, studies such as these should be excluded from evidence‐based reviews of ASI and rather be included in reviews of sensory‐based interventions as Case‐Smith et al. [2015] suggest.

It is imperative that in future studies of ASI, intervention procedures are manualized and monitored to ensure fidelity. Moreover, participants should be given a comprehensive assessment of sensory integration to confirm that they are appropriate candidates for this intervention, i.e., that difficulties processing and integrating sensation are impacting the behavior and functional skills of the children included in the study. A comprehensive assessment of sensory integration is also required so that interventionists can individually tailor the intervention to address each child's specific difficulties and strengths related to processing and integrating sensation and how these are impacting function.

Additional rigorous intervention studies are needed to determine whether ASI intervention is effective for children with other diagnoses or conditions, such as attention deficit disorder, mental retardation, history of adverse childhood experiences, or sensory integrative dysfunction with no other medical, developmental, or psychiatric diagnosis. One strategy for building this body of knowledge, beyond group comparison studies, is the use of methodologically sound single case experimental design (SCED) studies. SCEDs use rigorous research designs and are widely used across professions and across different types of interventions [Horner et al., 2005; Tate et al., 2016; Tate et al., 2016].

In practice, ASI intervention for children with autism is usually provided by occupational therapists who practice in multiple settings [May‐Benson et al., 2014]. In public special education settings in the United States, occupational therapy is classified as a related service that supports children's ability to benefit from educational services. ASI intervention is one approach that is often used to help achieve this aim. Our study shows that sufficient evidence supports the use of this approach. However, if an evidence‐based practice is desired, then the practice of ASI intervention must be consistent with the essential elements of this intervention, as described in the ASI Fidelity Measure. This is necessary to ensure that the intervention delivered is similar to the intervention that our study shows is an evidence‐based practice.

## Conclusion

Ayres Sensory Integration intervention is frequently requested by parents and is often utilized by occupational therapists for children with autism spectrum disorders, including those served in special education settings. The results of this systematic review indicate that it meets the criteria for an evidence‐based practice according to the CEC Standards for Evidence‐Based Practices in Special Education [Cook et al., 2015; Council for Exceptional Children [CEC], 2014]. It also appears to meet the criteria for an evidence‐based practice as defined by the United States Preventative Services Task Force [2012] and the FPG Child Development Institute Guidelines [Wong et al., 2015]. Consumers, third‐party payers, and professionals concerned with the well‐being of children with autism spectrum disorders can feel confident that ASI is an effective intervention for this population, particularly for those with IQs above 65 and who are 4–12 years of age. However, it is critical that therapists providing ASI intervention adhere to the essential elements of this intervention, to ensure that the intervention delivered is in keeping with an evidence‐based practice.

## Author contributions

SS: Participated in conceptualization of the paper, reviewed articles, collected and analyzed data, wrote, revised and edited all sections of the manuscript. SL: Participated in development and implementation of the systematic review, conducted initial article screening, contributed to article review data collection, data analysis, writing and editing manuscript and tables. ZM: Participated in the design of the review, reviewed articles, collected and analyzed data, wrote, revised and edited drafts of the manuscript. TMB: Participated in article review, data collection, data analysis, formulation of figure and tables, editing manuscript. LDP: Participated in review of articles, data collection, and data analysis. Participated in writing and editing the manuscript and tables. SSR: Participated in review of articles, data collection, and data analysis, edited sections of the manuscript. RS: Conceived of the idea for the paper, participated in article review, collected and analyzed data, wrote, revised and edited drafts of the manuscript. All authors read and approved the final manuscript.

## Conflict of interest

The authors report no conflict of interest.

## Compliance with ethical standards

The authors declare they have no conflict of interest. For this type of study, formal consent was not required.

## Supporting information


**Table S1:** Search Strategy used for Medline
