# Supplementary material for: A systematic review of ayres sensory integration intervention for children with autism
Source: Autism Res. 2018 Dec 12;12(1):6–19. doi: 10.1002/aur.2046 (PMC6590432; doi:10.1002/aur.2046)
Supplement: Supplementary file 1 — Table S1: Search Strategy used for Medline [file AUR-12-6-s001.docx]

Supplemental information: Search Strategy used for Medline

| Condition | Intervention | Study design | Additional limits |
| --- | --- | --- | --- |
| clumsy child syndrome*.mp. | exp Adaptation, Psychological/ or adaptive behavio?r*.mp. | appraisal.mp. | English language |
| developmental coordination disorder*.mp. | Ayres Sensory Integration.mp. | best practices.mp. | "all child (0 to 18 years)" |
| ((attention, motor and perception) adj3 disorder*).mp. | bilateral coordination.mp. | case control.mp. | Yr=”2006-Current” |
| developmental dyspraxia.mp. | emotional regulation.mp. | case report.mp. or Case Reports/ |  |
| gross motor deficit*.mp. | Executive Function/ | case series.mp. |  |
| exp Learning Disorders/ | Exercise/ | Practice Guidelines as Topic/ or clinical guideline*.mp. |  |
| (nonverbal learning disorder* or non verbal learning disorder*).mp. | motor planning.mp. | clinical trial.mp. or Clinical Trial/ |  |
| perceptual motor deficit*.mp. or exp Perceptual Disorders/ | (multisensory integration or multi sensory integration).mp. | Cohort Studies/ or cohort.mp. |  |
| regulatory disorder*.mp. | occupation based.mp. | Comparative Study/ |  |
| sensory integrative dysfunction*.mp. | Occupational Therapy/ | consensus development conference*.mp. |  |
| sensory modulation dysfunction*.mp. | ocular motor skill*.mp. | critique.mp. |  |
| sensory motor deficit*.mp. | "Play and Playthings"/ | (crossover or cross over).mp. |  |
| sensory processing disorder*.mp. | perceptual motor learning.mp. | Double-Blind Method/ or double blind.mp. |  |
| Sensory Deprivation/ | praxis.mp. | epidemiology.mp. or Epidemiology/ |  |
| asperger syndrome/ or autism spectrum disorder/ or autistic disorder/ | sensory diet.mp. | evaluation stud*.mp. or Evaluation Studies/ |  |
| pervasive developmental disorder*.mp. | (sensorimotor integration or sensory integration or SI).mp. | evidence based.mp. |  |
| autism.mp. | sensory integrative.mp. | evidence synthesis.mp. |  |
|  | Social Participation/ | Feasibility Studies/ or feasibility stud*.mp. |  |
|  | tactile stimulation.mp. | Follow-Up Studies/ or follow-up.mp. |  |
|  | touch pressure.mp. | health technology assessment.mp. |  |
|  | vestibular stimulation.mp. | intervention.mp. |  |
|  |  | Longitudinal Studies/ or longitudinal.mp. |  |
|  |  | main outcome measure.mp. |  |
|  |  | meta analysis.mp. or Meta-Analysis/ |  |
|  |  | Multicenter Studies as Topic/ or multicenter stud*.mp. |  |
|  |  | observational stud*.mp. |  |
|  |  | (outcome and process assessment).mp. |  |
|  |  | pilot.mp. |  |
|  |  | practice guideline*.mp. |  |
|  |  | Prospective Studies/ or prospective.mp. |  |
|  |  | random allocation.mp. |  |
|  |  | randomi?ed controlled trial*.mp. |  |
|  |  | Retrospective Studies/ or retrospective.mp. |  |
|  |  | sampling.mp. or Sampling Studies/ |  |
|  |  | scientific integrity review.mp. or "Scientific Integrity Review"/ |  |
|  |  | single subject design.mp. |  |
|  |  | standard of care.mp. or "Standard of Care"/ |  |
|  |  | systematic review.mp. |  |
|  |  | treatment outcome.mp. |  |
|  |  | validation stud*.mp. |  |
|  |  | multiple baseline.mp. |  |
|  |  | Cross-Sectional Studies/ or cross sectional.mp. |  |
